# Effects of neuroticism on pre-exam irritable bowel syndrome in female middle school students: mediating role of intolerance of uncertainty and moderating role of exercise duration

**DOI:** 10.3389/fpsyt.2024.1420970

**Published:** 2024-08-14

**Authors:** Hou Wu, Qiqin Liu, Jianping Liu, Mingfan Liu

**Affiliations:** ^1^ School of Psychology, Jiangxi Normal University, Nanchang, China; ^2^ College Counseling Center, Nanchang Institute of Technology, Nanchang, China; ^3^ Department of Culture and Tourism, Gaoan Secondary Specialized School, Yichun, China

**Keywords:** neuroticism, intolerance of uncertainty, irritable bowel syndrome, exercise time, female adolescents

## Abstract

**Background:**

China, which is deeply influenced by Confucianism, places special emphasis on students’ test scores. Previous studies have shown that neuroticism is associated with irritable bowel syndrome (IBS) in adolescents. However, the mechanisms underlying this association before exams in female secondary school students are unknown. The present study sought to ascertain whether IU mediates the association between neuroticism and pre-exam IBS, and to determine whether exercise duration moderates the relationship between neuroticism and pre-exam IBS.

**Methods:**

The sample consisted of 685 Chinese female middle school students (M_age_ = 14.81, SD = 1.55, range = 11-18) who completed paper questionnaires, including the neuroticism subscale of the Chinese Neuroticism Extraversion Openness Five-Factor Inventory, the IBS Symptom Severity Scale, a simplified version of the Intolerance of Uncertainty Scale, and a movement time questionnaire. Independent samples t-test was used to compare differences between groups and Pearson correlation coefficient was used to investigate the bivariate correlation. The SPSS PROCESS 4.1 plug-in was then used to examine the mediating role of IU as well as the moderating role of movement time between neuroticism and pre-exam IBS.

**Results:**

Neuroticism and IU were significantly correlated with pre-exam IBS (*r* = 0.39, 0.30, respectively; all *p* < 0.01), and neuroticism was significantly correlated with IU (*r* = 0.46, *p* < 0.01). Neuroticism had a direct predictive effect on pre-exam IBS in Chinese female middle school students (*β* = 0.32, *p* < 0.001), and IU also had a positive effect on pre-exam IBS (*β* = 0.15, *p* < 0.001). The mediating effect value of IU on the total effect was 18.09%. The relationship between neuroticism and pre-exam IBS was moderated by movement time (*β* = -0.23, *p* < 0.05).

**Conclusion:**

IU plays a mediating role between neuroticism and pre-exam IBS, and exercise time plays a moderating role between neuroticism and pre-exam IBS. These findings provide an evidence for neuroticism intervention, IU management, and pre-exam IBS improvement in female middle school students.

## 1 Introduction

“Officialdom is the natural outlet for good scholars.”

—The Analects of Confucius

The long-standing influence of Confucian cultural thinking subjects Chinese youth to tremendous pressure regarding studying and examinations. Approaching exams are the most stressful period for secondary school students, making them vulnerable to a range of physical and mental health problems, including irritable bowel syndrome (IBS) (1). IBS is a chronic gastrointestinal dysfunction in which patients experience abdominal pain and altered bowel habits, with the main symptoms being diarrhea, constipation, or both (2). Girls have higher levels of academic stress than boys and are more likely to experience negative emotions such as depression and anxiety (3). A meta-analysis of the prevalence of the IBS showed that the prevalence was 12.0% in women and 8.6% in men, with women being the group with the highest prevalence (4). A survey of Chinese students showed that IBS was more common in female students (11.3%) than in male students (10.3%) (5). The incidence of IBS in adolescents is increasing annually, and adolescents are considered a high-risk group (6). IBS causes inconvenience in female secondary school students’ lives and studies, causing invisible mental stress, which particularly affects their pre-exam preparation and exam performance. Moreover, the recurrent nature of IBS symptoms leads to significant consumption of medical resources and increases national healthcare costs (7). IBS is a multifactorial disorder of unknown origin with multiple risk factors, including genetics, diet (gluten intolerance, lactose malabsorption), gut microbiota, infectious gastroenteritis, psychological factors (anxiety, or depression) (8), other medical disorders, stress, sleep disorders (9), and personality factors (neuroticism) (10). High school students face test anxiety (11) and stress before exams, both of which may be triggers for the development of IBS. According to the psychophysiological model, IBS is a somatic manifestation of psychological problems, and anxiety in particular plays an important role in the development of IBS (12). According to the Selye stress model, stressors have an important impact on the body’s response and can lead to various adverse physical and psychological reactions, such as depression, anxiety, and gastrointestinal dysfunction (13). Studies have shown that stress can effectively predict IBS (14, 15). Stressful events can cause an imbalance in the regulation of an individual’s neuroendocrine-immune system, which acts on the intestinal tract through the brain-gut axis, leading to IBS (16, 17). Reducing stress, in turn, can help improve IBS symptoms (18). Therefore, female students are more likely to experience IBS before exams, which requires urgent attention. Thus, this study aimed to identify IBS’s underlying psychological mechanisms to enrich the study of the mechanism of IBS occurrence and to provide a theoretical basis for scientific prevention and intervention.

### 1.1 Neurotic personality and IBS

Secondary school is an important stage for the sound development of individual personality. Neuroticism increase the probability of psychosomatic diseases such as IBS (19, 20). Neuroticism may be an important predictor of IBS for the following considerations. On the one hand, the close correlation between neuroticism and IBS symptoms (7). Yastiba et al. found that neuroticism plays a role in IBS health-related quality of life through negative illness-related perceptions (19). Neuroticism has an indirect effect between traumatic experiences and functional gastrointestinal symptoms (21). On the other hand, neuroticism fits with the pathogenesis of IBS. Potential factors in the pathogenesis of IBS include psychological distress (22) and stress (23), and psychotherapy is beneficial in IBS (22). Neuroticism may fit into the pathogenesis of IBS by influencing an individual’s ability to process negative emotional distress and stress (24). Based on the biopsychosocial model, IBS is caused by the interaction of biological, psychological, environmental, and social factors associated with pain and dysfunction. Personality characteristics and emotions are the core components that affect IBS through a series of physiological and behavioral pathways (10), such as participating in the functions and disorders of the brain-gut axis and promoting the onset and recurrence of IBS (15, 25). Hence, exploring the mechanism of the role of neuroticism in adolescent IBS can help to better promote the physical and mental health of students. According to the risk enhancement model, individuals with IBS may develop stress and anxiety due to being afflicted with long-term disease, which can cause sympathetic excitation accompanied by metabolic disturbances, such as sodium and water retention or reduced catecholamine hormones, and exacerbate negative emotions (26), thus continuously exacerbating IBS symptoms. Empirical studies have shown that prognosis is affected by a prolonged IBS course, long-term negative emotions, and reduced resilience (27). In sum, neuroticism may be an important risk and predictor of IBS.

### 1.2 Intolerance of uncertainty and IBS

According to endogenous stress models, endogenous stressors of IBS include excessive avoidance and control issues in individuals with IBS symptoms and fear of recurrence of IBS symptoms (28). Endogenous stress is the key factor in the severity and recurrence of symptoms in patients with IBS, and changes in endogenous stress are the primary focus of IBS psychotherapy (29). This means that individuals do not have to be overly controlling or fearful of IBS symptoms, and being able to tolerate uncertainty contributes to the improvement of IBS symptoms and reduces recurrence. Intolerance of uncertainty (IU) refers to an individual’s inability to tolerate uncertain information, which can lead to a negative and unstable reaction and a persistent feeling of uncertainty (30). Individuals high in IU show more positive beliefs about worry (31), tend to have a negative problem orientation (32), and IU scale measures assess metacognitive beliefs (33); therefore, IU can be considered a metacognitive belief. Metacognitive theory suggests that psychological disorders arise and develop as a result of maladaptive coping patterns when individuals are confronted with negative thoughts or beliefs (34), and its focus on cognitive patterns is a perspective that facilitates psychopathology and intervention research. IU levels are likely to increase as cell phone and internet use increases (35). Uncertainty tolerance is a predictor of anxiety in youth (36) and of psychological distress tolerance in IBS patients (37). Krasner et al. found that uncertainty tolerance is an important factor influencing IBS and can be used to differentiate IBS subtypes (38). In contrast, it has also been noted that variability in individual uncertainty tolerance is not related to IBS (39). This finding is not consistent with previous findings on the relationship between IU and IBS. Thus, this study aimed to further explore the relationship between IU and IBS.

Furthermore, it has been shown that there is a significant positive correlation between neuroticism and IU (40). This means that individuals with high in neuroticism traits are more likely to be intolerant of uncertainty in ambiguous situations. Because exams are fraught with uncertainty, highly neurotic individuals are more likely to experience stress reactions. Therefore, highly neurotic individuals who encounter uncertain threat signals will use safety strategies more often to avoid more uncertain stimuli (41). In sum, based on endogenous stress models, worry about the onset of IBS, i.e. fear or anxiety about an uncertain event that has not occurred, can exacerbate IBS symptoms or recurrence.

### 1.3 The role of exercise duration in predicting IBS symptoms

Exercise is the foundation of health and is conducive to recovery from psychosomatic diseases (42). Studies have shown that exercise significantly reduces IBS symptoms (43). Long-term regular exercise can curb the worsening of IBS symptoms (44, 45). In a long-term follow-up study of IBS, subjects showed increased physical activity and improved symptom scores during follow-up compared with baseline (46), suggesting that physical activity intervention has a positive long-term effect on IBS and psychological symptoms. Exercise is an easy and effective way to treat IBS (43). This has a significant effect on the sports intervention program for teenagers’ physical and mental health, in which the exercise time is generally 30-50 minutes (47). Regular exercise interventions significantly improve the physiological state, mental health level, and environmental suitability of adolescents (47). It is worth noting that exercise time must be over 30 minutes (48). In addition, lower levels of neuroticism have been found in the population of people who exercise regularly (49), which may be the cause of the reduction in IBS symptoms. Therefore, this study predicted that exercise duration would be related to the improvement in IBS symptoms and that IBS symptoms would be less severe in the high exercise duration group and more severe in the low exercise duration group.

### 1.4 The present study

Previous studies on the relationship between neuroticism, IU and IBS remain controversial. Some studies have indicated that there is an association between neuroticism, IU and IBS (20, 38). However, some studies have shown inconsistent results, such as variability in individual uncertainty tolerance not being associated with IBS (39). These differences may be due to the variability of the study population or the duration of the study. Previous studies have also focused on single factors (e.g., neuroticism, IU) in association with IBS, while ignoring the interaction or joint effects of the two. Physical activity reduced the association between neuroticism and adverse cognitive outcomes (50) and improved IBS symptoms (43). Therefore, three variables, neuroticism, IU, and physical activity time, were selected to predict IBS in the present study. As neuroticism is positively associated with IBS (7, 19, 20) and there is a significant positive correlation between neuroticism and IU (40). According to endogenous stress models, concern about the uncertainty of IBS symptoms with excessive avoidance is a key factor in IBS recurrence (28). Therefore, we suggest that IU mediates the relationship between neuroticism and IBS. In the study of the mediator variable IU, the reporting guidelines for mediator analyses were referenced (51). Exercise improves negative mood in neurotic individuals and reduces symptoms of IBS (43–45). Therefore, we suggest that the relationship between neuroticism and IBS is moderated by the role of exercise.

Given the impact of exams on middle school students, there were some changes in the characteristics of IBS, neuroticism, and IU prior to the exams. Specifically, neuroticism and IU negatively affected individuals’ coping with exams, whereas physical activity positively affected coping with exams. Test anxiety and test stress exacerbated individual IBS symptoms. In summary, we chose female middle school students in the pre-exam context to model mediated regulation. The effects of neuroticism, IU, and exercise on IBS symptoms were explored in a population of secondary school students in a pre-exam context. This has important theoretical and practical implications for our targeted response to the challenges of exams and pre-exam IBS in secondary school students.

In conclusion, the objective of this study was to examine the interrelationships between neuroticism, IU, pre-exam IBS and exercise time. Specifically, the study aimed to test the mediating role of IU between neuroticism and pre-exam IBS and to investigate the moderating role of exercise time between neuroticism and pre-exam IBS.

## 2 Methods

### 2.1 Participants

The study employs a cross-sectional research design. The study was conducted in a classroom setting by administering questionnaires to the participants in their schools. Each school provided official approval, and prior informed consent was obtained from the participants and their guardians. The Human Research Ethics Committee of Jiangxi Normal University approved the recruitment and data collection procedures. The 10 research assistants were first trained by the principal investigator on how to read the instructions to the subjects, how to ensure that the subjects’ answers were anonymous and confidential, how to maintain a quiet environment during the test, and how to answer the subjects’ questions. The research assistants then went to the subjects’ classes to distribute the questionnaires. The study was conducted among female students from junior one to senior three who were normally enrolled in school, excluding those who were unable to study normally due to illness.

Using cluster random sampling method, female secondary school students from four schools in Jiangxi and Zhejiang provinces in China were surveyed. A total of 709 questionnaires were collected within 1 month before the final exam. The data set was filtered to exclude questionnaires that were not completed in full and responses that were obviously not serious. This included questionnaires completed by individuals whose age was obviously beyond the range of ordinary middle school students and those who chose the same option for all questions. This process yielded a final data set of 685 valid questionnaires, with a valid response rate of 96.61%.

The reliability of the mediated effects analysis in this study was tested using Monte Carlo Power Analysis for Indirect Effects (https://schoemanna.shinyapps.io/mc_power_med/) (52). When the sample size was set at the actual number of this study (N = 685) and the variable correlation coefficients were the actual results of this study, the power was found to be 0.97, indicating that the sample size for the mediation analysis in this study was adequate.

### 2.2 Measurements

#### 2.2.1 Neuroticism

The NEO Five-Factor Inventory was developed by Costa and McCrae, and the internal consistency ranges from 0.68 to 0.86 (53). Yao et al. revised the Chinese version of the Big Five personality scale (54), with the neuroticism subscale being selected for this study. The Cronbach’s α coefficient for the neurotic subscale was 0.77 (54). The 12 items are scored on a 5-point scale (1 = completely inconsistent to 5 = completely consistent). Higher scores on this scale indicate higher levels of neuroticism. In this study, the Cronbach’s α was 0.84.

#### 2.2.2 IBS

In order to gain a deeper understanding of the severity variation observed in individuals with IBS, Francis and colleagues developed the IBS symptom severity scale (IBS-SSS) (55). The IBS-SSS is scored on a 10-centimetre visual scale, exhibiting good reliability and validity (55). The scale encompasses five domains: the intensity and frequency of abdominal discomfort, the degree of abdominal distension, the nature of defecation, and the extent to which disease affects the individual’s quality of life. A total score of 75 or less is considered normal; a score of 75 to 175 is considered mild; a score of 175 to 300 is considered moderate; and a score of 300 or more is considered severe. In this study, the Cronbach’s α of the IBS-SSS scale was 0.70.

#### 2.2.3 IU

The IU scale was developed by Carleton et al., and demonstrated a Cronbach’s α of 0.91 (56). The Chinese version of the IU scale was revised by Wu Lijuan et al. with 12 items on a 5-point scale (1 = completely inconsistent to 5 = completely consistent), with a retest reliability of 0.80 (57). Higher scores on this scale are indicative of a reduced capacity to tolerate uncertainty. In this study, the Cronbach’s α coefficient was 0.82.

#### 2.2.4 Exercise time

A questionnaire was developed in this study to investigate the exercise time of female secondary school students before the examination. The questionnaire was based on the self-reported question “How many minutes do you spend exercising each day when you are feeling stressed?” In this study, less than 30 minutes was considered to represent low exercise time, while 30 minutes or more was considered to represent medium-to-high exercise time.

#### 2.2.5 Statistical analyses

The data analysis was completed using SPSS 25.0 and the PROCESS 4.1 plugin. The single factor test developed by Harman was employed to assess the potential for common method bias in the four variables under investigation. Descriptive statistical analysis is employed to ascertain the characteristics of the variables within the population. The bivariate correlation between neuroticism, uncertainty tolerance, pre-exam IBS and exercise time was investigated using an independent sample t-test and Pearson correlation coefficient. The variable data has been subjected to standardization. The SPSS PROCESS 4.1 plugin was employed for the purpose of conducting a mediated regulation analysis (58). The Model 4 examined the mediating role of uncertainty tolerance between neuroticism and pre-exam IBS. The mediating effect was examined using the bootstrap method with a sampling size of 5,000. Subsequently, the Model 5 was employed to assess the moderated mediation effect of exercise time grouping between neuroticism and pre-exam IBS. All tests were within the 95% confidence interval. If the confidence interval did not include zero, the mediation effect was significant at the 0.05 level.

## 3 Results

### 3.1 Common method variance

Harman’s one-way test with exploratory factor analysis was applied to the data, and it was found that there were seven factors with eigenvalues greater than 1. The first factor explained 24.18% of the variance, which is less than the critical threshold of 40%. Therefore, it can be concluded that the common method bias present in the study data was not significant.

### 3.2 Descriptive statistics and correlation analysis

The mean age of the participants was 14.81 years (standard deviation = 1.55, range 11–18). The study involved students from six grades, spanning from junior to senior high school (**Table 1**). Before the final exams, 249 students had mild IBS, 70 students had moderate IBS, and 5 students had severe IBS. The preliminary detection rate of IBS among students prior to the final examinations was 47.30%.

**Table 1:** Participants’ general characteristics.

Variable	Classification	Number of cases	Percentage (%)
Grade	Junior one	112	16.35
	Junior two	116	16.93
	Junior three	106	15.47
	Senior one	115	16.79
	Senior two	109	15.91
	Senior three	127	18.54
Exercise time	Low exercise time	590	86.13
	Medium high exercise time	95	13.87
Total		685	100

**Table 2** indicates that the two groups exhibited significant differences in total scores on the neuroticism, IU, and IBS scales. The results demonstrated that neuroticism, IU, and IBS were significantly higher in the IBS group than in the control groups (*p* < 0.001). No significant differences were observed between the two groups with regard to age and exercise time.

**Table 2:** Comparison of differences in variables between the female IBS and the control groups, and descriptive analysis results.

Variable	IBS group (n=324)	Control group (n=361)	t	p	M	SD	1	2	3	4	5
Age	14.84 ± 1.62	14.78 ± 1.49	0.517	0.605	14.81	1.55	1				
Neuroticism	36.77 ± 8.34	30.98 ± 8.09	9.213^***^	0.000	33.72	8.70	0.05	1			
IU	30.58 ± 8.07	26.29 ± 7.44	7.236^***^	0.000	28.32	8.03	-0.07	0.46^**^	1		
pre-exam IBS	141.20 ± 51.35	40.51 ± 23.92	33.435^***^	0.000	88.14	63.85	0.06	0.39^**^	0.30^**^	1	
Exercise time subgroup	1.16 ± 0.37	1.12 ± 0.32	1.565	0.118	1.14	0.35	-0.03	-0.02	0.07	0.04	1

**p < 0.01, ***p < 0.001, Exercise time subgroup was dummy coded such that 0 = low exercise time and 1 = medium to high exercise time.

The descriptive statistics for neuroticism, IU, pre-exam IBS, and exercise time are presented in **Table 2**. The correlation between neuroticism and pre-exam IBS was found to be significantly positive, while the correlation between neuroticism and IU was also found to be significantly positive. A significant positive correlation was observed between IU and pre-exam IBS. The correlation between neuroticism, IU and pre-exam IBS and the exercise time groupings was not statistically significant.

### 3.3 The mediating effect of IU

**Figure 1:**
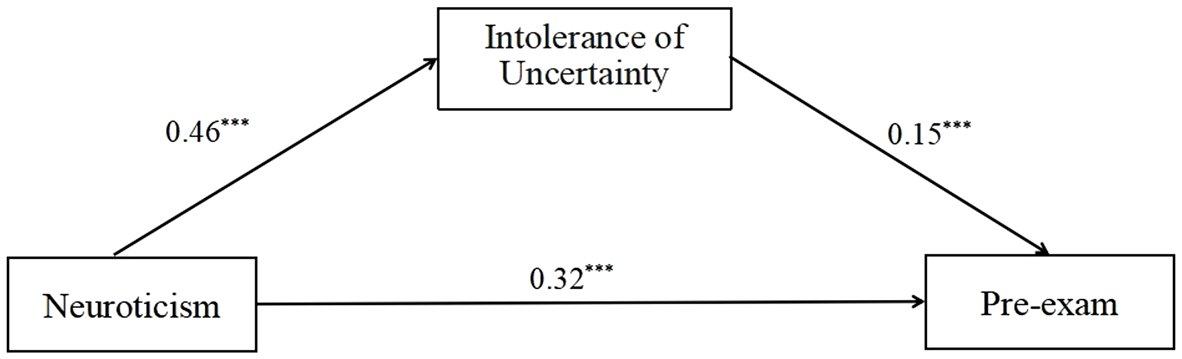
Mediating role of intolerance of uncertainty between neuroticism and pre-exam IBS. The results showed that intolerance of uncertainty can significantly improve pre-exam IBS. ***p < 0.001.

The correlation analysis of neuroticism, IU, and IBS in middle school female participants revealed a significant correlation (*p* < 0.01), which permitted the testing of the mediating effect of IU on the relationship between neuroticism and IBS. In order to test the mediating effect, the SPSS PROCESS 4.1 plugin was employed, and the results are presented in **Table 3**, **Figure 1**. With neuroticism as the independent variable, pre-exam IBS as the dependent variable, and IU as the mediating variable, Model 4 shows that neuroticism has a significant positive impact on female secondary school students’ pre-exam IBS (*β* = 0.32, *p* < 0.001). The results indicate that IU has a significant positive impact on pre-exam IBS (*β* = 0.15, *p* < 0.001). A significant positive effect was observed for neuroticism on IU (*β* = 0.46, *p* < 0.001).

**Table 3:** The mediating role of IU between Neuroticism and pre-exam IBS.

Outcome variable	Factor	β	SE	t	LLCI	LLCI
Pre-exam IBS	Neuroticism	0.32	0.04	8.04^***^	0.24	0.39
	IU	0.15	0.04	3.85^***^	0.07	0.23
IU	Neuroticism	0.46	0.03	13.64^***^	0.40	0.53

***p < 0.001.

A bootstrap method was employed for 5,000 repeated samplings to assess the mediating effect of IU. The results demonstrated that the indirect effect of neuroticism on pre-exam IBS through IU did not include zero in the 95% confidence interval (CI = [0.03, 0.12]), indicating that the mediating effect of IU was significant. The results indicated that IU exerted a partial significant mediating effect on the relationship between neuroticism and IBS. The mediating effect was 0.07, while the direct effect was 0.32. The ratio of the mediating effect to the overall effect was 18.09%.

### 3.4 The moderating effect of exercise time

**Figure 2:**
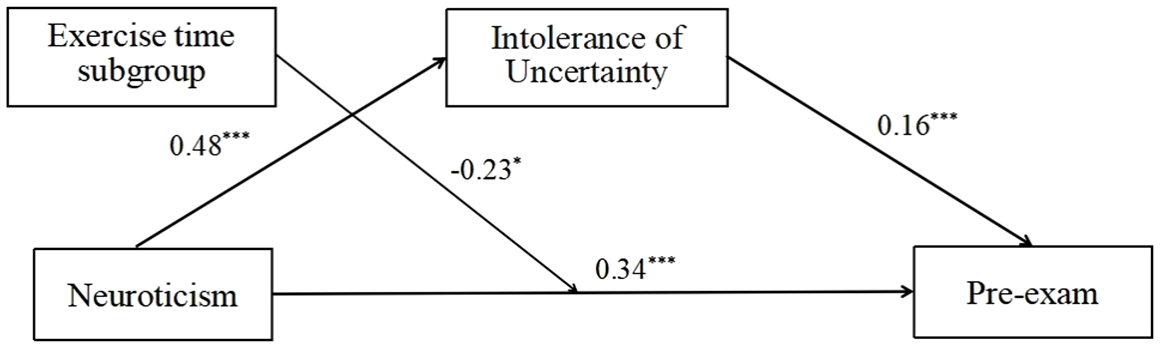
Intolerance of uncertainty plays a mediating role between neuroticism and pre-exam IBS. At the same time, exercise time can play a regulatory role in the direct path. The results showed that neuroticism can increase the level of pre-exam IBS, but exercise can significantly inhibit this effect. *p < 0.05; ***p < 0.001.

The moderating effect of exercise time on neuroticism and pre-exam IBS was tested using Model 5, with the results presented in **Table 4**, **Figure 2**. The product of neuroticism and exercise time subgroup exhibited a significant predictive effect on pre-exam IBS (*β* = -0.23, *t* = -2.21, *p* < 0.05), indicating that exercise time played a moderating role in the prediction of pre-exam IBS by neuroticism. The confidence interval of the model test does not include zero (CI = [-0.44, -0.03]), indicating that the moderated mediating effect is significant.

**Table 4:** Moderated mediating effect of exercise time subgroup on the influence of neuroticism on pre-exam IBS.

Outcome variable	Factor	β	SE	t	LLCI	LLCI
Pre-exam IBS	Neuroticism	0.34	0.04	8.11^***^	0.26	0.43
	IU	0.16	0.04	3.95^***^	0.08	0.24
	Exercise time subgroup	0.10	0.10	0.93	-0.11	0.30
	Neuroticism × Exercise time subgroup	-0.23	0.10	-2.21^*^	-0.44	-0.03
	Age	0.03	0.06	0.51	-0.09	0.15
	Grade	0.002	0.05	0.04	-0.10	0.11

*p < 0.05, ***p < 0.001.

To gain further insight into the moderating effect of exercise time on the relationship between neuroticism and pre-exam IBS in female secondary school students, a simple effect analysis was conducted. The results indicated that neuroticism was a significant predictor of pre-exam IBS in the group with low exercise time (*β*_simple_ = 0.34, *t* = 8.11, *p* < 0.001), while the predictive effect was not significant in the group with moderate or high exercise time (*β*_simple_ = 0.11, *t* = 1.12, *p* > 0.05). This indicates that the impact of neuroticism on pre-exam IBS is less pronounced in individuals with moderate to high exercise duration. In conclusion, the moderated mediation model proposed in this study has been empirically validated. The results indicate that IU plays a mediating role between neuroticism and pre-exam IBS. Furthermore, the influence of neuroticism on pre-exam IBS is regulated by exercise time.

## 4 Discussion

To the best of our knowledge, this study is the first to focus on the link between neuroticism and pre-exam IBS among Chinese adolescent females. Our results indicated that the prevalence of pre-exam IBS among Chinese female students was very high. In addition, individuals with high neuroticism were more likely to experience pre-exam IBS, which was mediated by IU. Furthermore, exercise duration moderated the direct relationship between neuroticism and pre-exam IBS. In this study, the prevalence of pre-exam IBS in female secondary school students was 47.30%, which was much higher than the prevalence in similar groups in Asia (7). This may be due to the fact that the participants in this study were Chinese adolescent females who were deeply influenced by Confucian cultural thinking. The prevalence of IBS was significantly higher in adolescent females than males, which is consistent with the findings of a large sample of studies (59). However, this may be related to the time frame chosen for our study, which was 1 month before the examination. Female secondary school students are more likely to develop IBS symptoms during the stressful period before exams, consistent with the stress response model, in which individuals are prone to develop general adaptation syndromes when under stress (13). IBS is an adverse reaction to long-term stress in female middle school students. Previous studies have shown that female students with IBS have significantly higher stress levels than female students without IBS (60). Females are a risk factor for IBS (61). With increasing competition and exam pressure, IBS will become more serious for female secondary school students, and their quality of life will continue to decline. Hence, early detection and intervention are particularly important for female secondary school students. Mindfulness-based stress reduction and gastrointestinal microbiota are important areas to explore for IBS interventions (62, 63).

As shown in **Table 2**, there was no significant differences in terms of age, indicating that age may not be a risk factor for IBS in adolescents. However, the results may differ when participants are outside a certain age range; for example, the incidence of IBS differs significantly between children and adolescents (59). We found a significant positive association between neuroticism and pre-exam IBS among female secondary school students. Neuroticism is an important risk factor and predictor of IBS (29). Previous studies have also shown a strong genome-wide correlation between IBS risk and neuroticism (64). Patients with IBS accompanied by neuroticism may perceive IBS as unpredictable and uncontrollable (19). A longitudinal empirical study showed that personality traits can be changed through psychological intervention (65). Therefore, psychological factors related to personality should be considered during IBS treatment, and it is meaningful to conduct comprehensive interventions with psychotherapy and personality refinement.

The present study suggests that IU mediates the relationship between neuroticism and pre-exam IBS. High neuroticism affects an individual’s IU, and high IU may increase IBS symptom severity. The results are in line with the endogenous stress model (28), in which an individual’s endogenous stress (low uncertainty tolerance) is an important mediator of symptom severity in individuals with IBS. This study provides empirical evidence for a physiopsychosocial model of IBS, in which personality traits and affective patterns influence IBS through a range of physiological and behavioral pathways (10); that is, neuroticism is the antecedent variable of IBS and acts on IBS by influencing an individual’s uncertainty tolerance (40). Physiopsychosocial influences, such as external adverse events, stress, and genetic susceptibility, can affect an individual’s physical and psychological growth and lead to the development of undesirable personality traits in adulthood, such as neurotic personality. Interaction via the brain-gut axis leads to IBS (25). Therefore, focusing on neuroticism and IU is beneficial for IBS prevention, diagnosis, and treatment. A recent study has shown that females and IU are associated with somatic symptoms (66), suggesting that IBS may have similar psychological influences as somatic symptoms. Improving IU levels may be a breakthrough for future interventions for IBS and somatic symptoms, such as using cognitive bias modification paradigms (67).

In this study, exercise duration moderated the effect of neuroticism on IBS symptom severity in female secondary school students. Specifically, neuroticism was a more significant predictor of IBS symptom severity in middle school girls with low physical activity levels than in those with high physical activity levels. This is consistent with the societal understanding that exercise is beneficial for physical and mental health. In addition, moderate exercise intervention had a therapeutic effect on IBS, consistent with the results of previous empirical studies (43). The reviewed intervention studies also confirmed that physical activity can improve physical, psychological, and academic performance in children and adolescents (48). In an intervention study, moderate to vigorous physical activity was defined as 30 minutes of exercise (68). Exercise duration is key to the effects of exercise, and the results of this study showed that an exercise duration of 30 minutes or more significantly suppressed IBS symptoms in female middle school students. Moreover, exercise increases the diversity and abundance of gut microbes, which improves IBS symptoms through the microbiome-gut-brain axis (69). Additionally, the process of improving IBS symptoms is influenced by psychological factors. For instance, exercise interventions can attenuate the effects of anxiety and depression on individuals and improve quality of life (19) and academic performance (48), which, in turn, alleviates excessive worry about IBS and exams in female secondary school students, thus forming a positive interaction. At the same time, exercise improves an individual’s resilience (70), which in turn may reduce the severity of IBS symptoms (71).

The results of this study have several important clinical applications for IBS. Firstly, in identifying and intervening with high-risk groups, special attention should be paid in clinical practice to the group of adolescents with neurotic traits, especially female students. For this group of students, schools and families should provide psychological support and emotional management to reduce pre-exam stress and feelings of uncertainty, which in turn reduces the risk of IBS. Secondly, the mediating role of IU, which focuses on female students’ acceptance of the uncertain outcomes of exams and illness and the development of tolerance, may help to reduce IBS symptoms. Increasing anticipation of disease outcomes through cognitive behavioral therapy may improve quality of life in people with IBS (19). Third, the moderating effect of exercise time, students should exercise regularly, especially maintain at least 30 minutes of exercise time, which may help in the treatment and alleviation of IBS before exams. In conclusion, improving students’ perception of uncertain events and moderate-to-vigorous physical activity should be incorporated into cognitive-behavioral therapy and the development of individualized intervention programmes for patients with IBS.

### 4.1 Limitations

This study has several limitations. First, the sample used in this study only included female secondary school students from two provinces in mainland China, so the limitations of the sample should be considered when generalizing the results of the study. Second, the present study was a cross-sectional investigation that could not infer causal relationships between the study variables. Future longitudinal studies are needed to assess IBS and its predictors in female secondary school students before exams. Third, Chinese female students were more negatively affected by exams than male students (72). Cultural factors should be taken into account when discussing the relationships between the variables in this study.

### 4.2 Conclusion

There was a significant positive correlation between neuroticism and pre-exam IBS; a significant positive correlation between neuroticism and IU; and a significant positive correlation between IU and pre-exam IBS. The mediating role of IU between neuroticism and pre-exam IBS. Exercise time moderated the effect of neuroticism on pre-exam IBS. The clinical applications of this study include a special focus on a group of adolescent females with neurotic traits, with the aim of reducing their risk of IBS by reducing pre-exam stress through psychological support and emotional management. Furthermore, the use of cognitive-behavioral therapy is recommended to help patients accept and tolerate the uncertain outcome of the disease. Finally, it is advised that students be encouraged to exercise regularly for at least 30 minutes as a means of alleviating pre-exam IBS symptoms.

## Data Availability

The original contributions presented in the study are included in the article/**Supplementary Material**. Further inquiries can be directed to the corresponding author.
